# An energy-reduced dietary pattern, including moderate protein and increased nonfat dairy intake combined with walking promotes beneficial body composition and metabolic changes in women with excess adiposity: a randomized comparative trial

**DOI:** 10.1002/fsn3.231

**Published:** 2015-04-13

**Authors:** Julie D Shlisky, Carrie M Durward, Melissa K Zack, Carolyn K Gugger, Jessica K Campbell, Sharon M Nickols-Richardson

**Affiliations:** 1 New York, New York, 10007; 2 Department of Nutrition, Dietetics and Food Sciences, Utah State UniversityLogan, Utah, 84322; 3 Clearinghouse for Military Family Readiness, The Pennsylvania State UniversityUniversity Park, Pennsylvania, 16802; 4 The Bell Institute of Health and Nutrition, General Mills, Inc., JFB Technical Center9000 Plymouth Ave N, Minneapolis, Minnesota, 55427; 5 Department of Food Science and Human Nutrition, The University of Illinois260A Bevier Hall, 905 S. Goodwin, Urbana, Illinois, 61801

**Keywords:** Body weight, dietary intervention, obesity, overweight, weight loss, weight loss maintenance

## Abstract

Moderate protein and nonfat dairy intake within an energy-reduced diet (ERD) may contribute to health benefits achieved with body weight (BW) loss. The current study examined the effectiveness of a weight-loss/weight-loss maintenance intervention using an ERD with moderate dietary protein (30% of kcals) and increased nonfat dairy intake (4–5 svg/d), including yogurt (INT group) and daily walking compared to an ERD with standard protein (16–17% of kcals) and standard nonfat dairy intake (3 svg/d) (COM group) with daily walking. A randomized comparative trial with 104 healthy premenopausal women with overweight/obesity was conducted in a university setting. Women were randomized to INT group or COM group. Anthropometric measurements, as well as dietary intake, selected vital signs, resting energy expenditure, blood lipids, glucose, insulin, and selected adipose-derived hormones were measured at baseline, and weeks 2, 12, and 24. Targets for dietary protein and nonfat dairy intake, while initially achieved, were not sustained in the INT group. There were no significant effects of diet group on anthropometric measurements. Women in the INT group and COM group, respectively, reduced BW (−4.9 ± 3.2 and −4.3 ± 3.3 kg, *P* < 0.001) and fat mass (−3.0 ± 2.2 and −2.3 ± 2.3 kg, *P* < 0.001) during the 12-week weight-loss phase and maintained these losses at 24 weeks. Both groups experienced significant decreases in body mass index, fat-free soft tissue mass, body fat percentage, waist and hip circumferences and serum triglycerides, total cholesterol, and leptin (all *P* < 0.001). Healthy premenopausal women with excess adiposity effectively lost BW and fat mass and improved some metabolic risk factors following an ERD with approximately 20% protein and 3 svg/d of nonfat dairy intake.

## Introduction

Weight loss achieved by following an energy-restricted dietary pattern promotes metabolic benefits (Fontana et al. 2007; Coppola et al. 2009; Lefevre et al. 2009). In addition to favorable outcomes conferred by reduced body weight (BW), research suggests additive beneficial effects of certain dietary components on various weight-related outcomes including body composition, insulin sensitivity, and risk factors for cardiovascular disease (Parker et al. 2002; Appel et al. 2003; Layman et al. 2003a).

Dietary protein intake has been widely studied, and benefits beyond BW loss have been reported with typical energy-restricted diets (Layman et al. 2008; Abete et al. 2010). Higher protein diets have resulted in enhancement of BW (Skov et al. 1999; Brehm and D'Alessio 2008) and fat mass losses (Layman et al. 2008, 2009), preservation of fat-free soft tissue mass (Leidy et al. 2007; Brehm and D'Alessio 2008) and improvement in blood lipids (Skov et al. 1999; Farnsworth et al. 2003; Layman et al. 2003b, 2005, 2009; Noakes et al. 2005). These results have been partially attributed to an increased thermic effect of food (Lejeune et al. 2006), increased satiety (Lejeune et al. 2006; Veldhorst et al. 2008) and maintenance of fat-free soft tissue mass (Layman et al. 2003b).

Similarly, increased dairy intake has been implicated in body composition improvements (5–10% decrease in fat mass) with corresponding BW loss of 5–10% (Zemel et al. 2005, 2009; Josse et al. 2011). Several studies have shown an inverse relationship between total dairy intake and body mass index (Pereira et al. 2002), nonfat dairy intake and fat mass (Zemel et al. 2005), body fat percentage (Poddar et al. 2009), truncal fat (Poddar et al. 2009) and waist circumference (Newby et al. 2003), and calcium intake and body fat percentage (Zemel et al. 2000). Dietary calcium (Zemel et al. 2004) as well as the branched-chain amino acid leucine (Koopman et al. 2005; Layman and Walker 2006) found in dairy products have been regarded as contributors to the reported link between dairy food consumption and weight-related outcomes. Specifically, nonfat yogurt combined with energy restriction has been shown to produce greater fat mass losses (∼4.5 kg) than energy restriction alone (∼2.8 kg) (Zemel et al. 2005). Healthy premenopausal women reduced fat mass and body fat percentage and increased fat-free soft tissue mass to a greater extent when consuming an energy-restricted diet with 30% of energy as protein and 6–7 servings of low-fat dairy per day with exercise, compared to women consuming an energy-restricted diet with adequate protein (15% of kcal) and 3–4 servings of low-fat dairy per day with exercise or adequate protein and <1 serving of low-fat dairy per day with exercise (Josse et al. 2011). Similarly, adults consuming roughly 30% of energy from protein who walked 5 days per week over a 4-month weight-loss period lost more fat mass and maintained that loss over 1 year, compared to adults consuming a conventional weight-loss diet with lesser protein (Layman et al. 2009). The higher protein group also experienced a greater reduction in triglycerides and elevation of high-density lipoprotein cholesterol concentrations (Layman et al. 2009).

Given that many of the previous studies have been cross-sectional, very short-term, laboratory-based feeding trials and/or not tested in free-living women (Skov et al. 1999; Parker et al. 2002; Farnsworth et al. 2003; Layman et al. 2003a,b; Noakes et al. 2005; Lejeune et al. 2006; Leidy et al. 2007; Zemel et al. 2009; Josse et al. 2011), the current study evaluated effects of moderate dietary protein and increased nonfat dairy intake as part of an energy-reduced diet (ERD) within a 6-month comprehensive lifestyle intervention in free-living premenopausal women with overweight/obesity. It was hypothesized that an ERD including moderate protein (30% of kcals) and increased nonfat dairy intake (4–5 svg/d) combined with daily walking (INT group) would induce greater reductions in BW, body mass index, fat mass, body fat percentage, waist and hip circumferences and maintenance of fat-free soft tissue mass, compared to an ERD, including standard protein (16–17% of kcals) and standard nonfat dairy intake (3 svg/d) (i.e., control diet) combined with daily walking (COM group), during 12 weeks of weight loss followed by 12 weeks of weight-loss maintenance. Effects on selected vital signs (i.e., resting heart rate and blood pressure), serum lipids, glucose and insulin concentrations, and selected adipose-derived hormones were further explored.

## Methods

### Participants, screening, enrollment and informed consent

**Figure 1:**
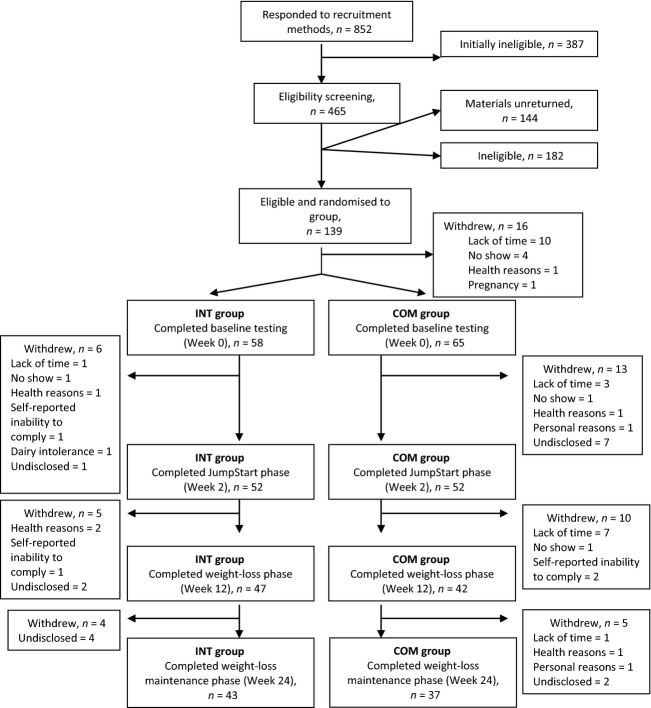
Diagram of recruitment, enrollment, randomization, and attrition of participants in a study examining changes in body composition and metabolic parameters in premenopausal women with overweight/obesity following an energy-reduced dietary pattern (ERD), including moderate protein and increased nonfat dairy intake(INT group) or an ERD with standard protein and standard nonfat dairy intake(COM group) combined with walking. Primary reasons for study withdrawal included lack of time (*n* = 22), undisclosed (*n* = 16), and no return for subsequent testing (*n* = 7).

Participants were recruited by newsletter, newspaper and radio advertisements, posted flyers, mailed advertisements, and by word-of-mouth. Selection criteria included: women aged 20–45 years with a body mass index of ≥25 and ≤36 kg/m^2^; limited physical activity (≤2 h of planned exercise/week); eumenorrhea; stable BW during the past 6 months; a score of <50 on the Zung Self-Rating Depression Scale/Status Inventory (Zung 1972); absence of yogurt intolerance, aversion, or allergy; consumption of <24 oz. of yogurt per day, ≤16 oz. of caffeinated beverages per day; ≤1 alcoholic beverage per day (i.e., ≤12 fl. oz. of regular beer or ≤5 fl. oz. wine or ≤1.5 fl. oz. 80-proof distilled spirits); and a desire to lose weight. Exclusion criteria included current smoking; pregnancy; diagnosed metabolic or health conditions; use of medications or supplements for metabolic or health conditions or for BW loss; health or orthopedic conditions limiting musculoskeletal activity; gastric bypass surgery; hysterectomy and ovariectomy without hormone replacement therapy; and/or oral contraceptive use of <2 years duration (if used). A flow diagram of response, screening, randomization and attrition throughout the study is displayed in Figure1.

This study was approved by the Institutional Review Board for Research Involving Human Subjects at The Pennsylvania State University, University Park, PA. All participants provided implied consent for initial screening and written informed consent before review of complete eligibility screening materials.

### Experimental approach and intervention

This randomized intervention was conducted from December 2009 to March 2011. Participants were stratified by baseline age, body mass index and self-reported physical activity and then randomized, using a random number generator, into one of two groups. Women in each group completed the JumpStart phase (weeks 0–2), the weight-loss phase (weeks 3–12; total of 12 weeks), and the weight-loss maintenance phase (weeks 13–24; total of 12 weeks).

Women in the intervention (INT) group followed an ERD (1200 kcal/d) that included nonfat yogurt (Yoplait Light®, General Mills, Inc., Minneapolis, MN) as part of two standard meals per day (breakfast and lunch) during the JumpStart phase. Each standard meal consisted of one, 6 oz. nonfat yogurt choice (100 kcal), one cup cereal (100–120 kcal), or one granola bar (90–95 kcal) (Nature Valley, General Mills, Inc.), and one fruit serving. For dinner, women were instructed to consume foods based on an exchange system (American Diabetes Association and American Dietetic Association 2008). Women in the INT group were instructed to consume an additional three servings of nonfat dairy intake each day, for a total of five servings per day. During the weight-loss phase, women in the INT group consumed an ERD (1500 or 1700 kcal/d) that included nonfat yogurt as part of a standard meal for breakfast and nonfat yogurt as a snack for a total of 4–5 servings of nonfat dairy intake per day. During the weight-loss maintenance phase, women in the INT group consumed an energy-balanced diet, adjusted for current BW that included nonfat yogurt twice each day for a total of 4–5 servings of nonfat dairy per day. Women in the INT group were provided with weekly supplies of nonfat yogurt, cereal, and granola bars throughout the study.

Women in the comparison (COM) group followed an ERD (1500 or 1700 kcal/d, based on individual estimated energy needs) (Mifflin et al. 1990) during the JumpStart phase. Women in the COM group were instructed to consume a total of three servings of nonfat dairy intake per day. During the weight-loss phase, women in the COM group continued their assigned ERD (1500 or 1700 kcal/d), with three servings of nonfat dairy intake per day. During the weight-loss maintenance phase, women in the COM group consumed an energy-balanced diet, adjusted for current BW, with three servings of nonfat dairy intake per day. Women in the COM group were instructed not to consume yogurt throughout the entire study.

For both groups, total energy was divided into food group exchanges, and women were instructed to follow their exchange patterns (Table1). By design, the intervention (INT group) was expected to produce a slightly greater energy deficit compared to the COM group during the JumpStart phase. Proportions of macronutrients are provided in Table1. It was expected that the ERD combined with walking would decrease BW by around one pound per week. All women were provided with dietary patterns, sample menus and exchange list information (American Diabetes Association and American Dietetic Association 2008).

**Table 1:** Food exchange patterns by group during the JumpStart, weight-loss and weight-loss maintenance phases in a study examining changes in body composition and metabolic parameters with an energy-reduced dietary pattern (ERD) including moderate protein and increased non-fat dairy intake (INT group) or an ERD with standard protein and standard nonfat dairy intake (COM group), combined with walking

Energy-level^a^ (Macronutrient distribution)	Pattern^b^^,^^c^	Intervention (INT) group combined with walking^d^ (*n* = 52)			Comparison (COM) group combined with walking (*n* = 52)	
		1200 kcal/d (29% PRO, 24% FAT, 47% CHO)	1500 kcal/d (30% PRO, 25% FAT, 45% CHO)	1700 kcal/d (30% PRO, 25% FAT, 45% CHO)	1500 kcal/d (17% PRO, 24% FAT, 59% CHO)	1700 kcal/d (16% PRO, 25% FAT, 59% CHO)
Baseline Testing: Anthropometric and body composition measurements; selected vital signs; serum lipids, glucose and insulin concentrations; selected adipose-derived hormones						
JumpStart Phase (Weeks 0–2)	BREAKFAST					
	Standard Meal^e^	1	–	–	0	0
	EXCHANGES					
	Dairy					
	Nonfat	1 (nonfat yogurt)^f^	–	–	1^g^^,^^h^	1^g^^,^^h^
	1% fat	0	–	–	0	0
	2% fat	0	–	–	0	0
	Starch	1 (select from list)^i^	–	–	1	2
	Fruit	1	–	–	1	1
	Vegetable	0	–	–	0	0
	Meat					
	Lean	0	–	–	0	0
	Medium fat	0	–	–	0	0
	Fat	0	–	–	0	0
	LUNCH					
	Standard Meal	1	–	–	0	0
	Exchanges					
	Dairy					
	Nonfat	1 (nonfat yogurt)^f^	–	–	1	1
	1% fat	0	–	–	0	0
	2% fat	0	–	–	0	0
	Starch	1 (select from list)^i^	–	–	2	2
	Fruit	1	–	–	1	1
	Vegetable	0	–	–	2	2
	Meat					
	Lean	0	–	–	0	0
	Medium fat	0	–	–	1	1
	Fat	0	–	–	2	2
	SNACK					
	Standard Meal	0	–	–	0	0
	Exchanges					
	Dairy					
	Nonfat	1^h^	–	–	0	0
	1% fat	0	–	–	0	0
	2% fat	0	–	–	0	0
	Starch	0	–	–	1	1
	Fruit	0	–	–	1	1
	Vegetable	2	–	–	0	1
	Meat					
	Lean	0	–	–	0	0
	Medium fat	0	–	–	0	0
	Fat	0	–	–	0	0
	DINNER					
	Standard Meal	0	–	–	0	0
	Exchanges					
	Dairy					
	Nonfat	2^h^	–	–	1	1
	1% fat	0	–	–	0	0
	2% fat	0	–	–	0	0
	Starch	0	–	–	2	3
	Fruit	0	–	–	1	0
	Vegetable	2	–	–	2	3
	Meat					
	Lean	6	–	–	0	0
	Medium fat	0	–	–	2	2
	Fat	1	–	–	2	3
Week 2 Testing: Anthropometric measurements						
Weight-Loss Phase (Weeks 2–12)	BREAKFAST					
	Standard Meal	–	1	1	0	0
	Exchanges					
	Dairy					
	Nonfat	–	1 (nonfat yogurt)^f^	1 (nonfat yogurt)^f^	1^g^^,^^h^	1^g^^,^^h^
	1% fat	–	0	0	0	0
	2% fat	–	0	0	0	0
	Starch	–	1 (select from list)^i^	1 (select from list)^i^	1	2
	Fruit	–	1	1	1	1
	Vegetable	–	0	0	0	0
	Meat					
	Lean	–	0	0	0	0
	Medium fat	–	0	0	0	0
	Fat	–	0	0	0	0
	LUNCH					
	Standard Meal	–	0	0	0	0
	Exchanges					
	Dairy					
	Nonfat	–	1^h^	1^h^	1	1
	1% fat	–	0	0	0	0
	2% fat	–	0	0	0	0
	Starch	–	1	1	2	2
	Fruit	–	1	1	1	1
	Vegetable	–	1	2	2	2
	Meat					
	Lean	–	3	3	0	0
	Medium fat	–	0	0	1	1
	Fat	–	1	1	2	2
	SNACK					
	Standard Meal	–	0	0	0	0
	Exchanges					
	Dairy					
	Nonfat	–	1 (nonfat yogurt)^f^	1 (nonfat yogurt)^f^	0	0
	1% fat	–	0	0	0	0
	2% fat	–	0	0	0	0
	Starch	–	0	0	1	1
	Fruit	–	1	1	1	1
	Vegetable	–	1	0	0	1
	Meat					
	Lean	–	3	3	0	0
	Medium fat	–	0	0	0	0
	Fat	–	0	0	0	0
	DINNER					
	Standard Meal	–	0	0	0	0
	Exchanges					
	Dairy					
	Nonfat	–	1^h^	2^h^	1	1
	1% fat	–	0	0	0	0
	2% fat	–	0	0	0	0
	Starch	–	0	1	2	3
	Fruit	–	0	0	1	0
	Vegetable	–	2	2	2	3
	Meat					
	Lean	–	0	0	0	0
	Medium fat	–	3	3	2	2
	Fat	–	2	3	2	3
Week 12 Testing: Anthropometric and body composition measurements; selected vital signs; serum lipids, glucose and insulin concentrations; selected adipose-derived hormones						
Weight-Loss Maintenance Phase (Weeks 13–24)	BREAKFAST					
	Standard Meal	–	0	0	0	0
	Exchanges					
	Dairy					
	Nonfat	–	1^j^	1^j^	1^g^^,^^h^	1^g^^,^^h^
	1% fat	–	0	0	0	0
	2% fat	–	0	0	0	0
	Starch	–	1	1	1	2
	Fruit	–	1	1	1	1
	Vegetable	–	0	0	0	0
	Meat					
	Lean	–	0	0	0	0
	Medium fat	–	0	0	0	0
	Fat	–	0	0	0	0
	LUNCH					
	Standard Meal	–	0	0	0	0
	Exchanges					
	Dairy					
	Nonfat	–	1^j^	1^j^	1	1
	1% fat	–	0	0	0	0
	2% fat	–	0	0	0	0
	Starch	–	1	1	2	2
	Fruit	–	1	1	1	1
	Vegetable	–	1	2	2	2
	Meat					
	Lean	–	3	3	0	0
	Medium fat	–	0	0	1	1
	Fat	–	1	2	2	2
	SNACK					
	Standard Meal	–	0	0	0	0
	Exchanges					
	Dairy					
	Nonfat	–	1^j^	1^j^	0	0
	1% fat	–	0	0	0	0
	2% fat	–	0	0	0	0
	Starch	–	0	0	1	1
	Fruit	–	1	1	1	1
	Vegetable	–	1	0	0	1
	Meat					
	Lean	–	3	3	0	0
	Medium fat	–	0	0	0	0
	Fat	–	0	0	0	0
	DINNER					
	Standard Meal	–	0	0	0	0
	Exchanges					
	Dairy					
	Nonfat	–	1^j^	2^j^	1	1
	1% fat	–	0	0	0	0
	2% fat	–	0	0	0	0
	Starch	–	0	1	2	3
	Fruit	–	0	0	1	0
	Vegetable	–	2	2	2	3
	Meat					
	Lean	–	0	0	0	0
	Medium fat	–	3	3	2	2
	Fat	–	2	2	2	3
Week 24 Testing: Anthropometric and body composition measurements; selected vital signs; serum lipids, glucose and insulin concentrations; selected adipose-derived hormones						

PRO, dietary protein; FAT, dietary fat; CHO, dietary carbohydrate.

^a^Energy-level included 1200 kcal/d in the INT group during the JumpStart phase only; 1500 kcal/d or 1700 kcal/d based on individual estimated energy needs during the weight-loss and weight-loss maintenance phases; energy-reduced diet included 500 fewer kcal/d than estimated needs. ^b^Pattern based on exchange system (American Diabetes Association and American Dietetic Association 2008); ^c^Calcium and vitamin D normalized to 1700 kcal/d INT group; supplementation designed to meet targets of 1500 mg of calcium and 10–20 *μ*g of vitamin D per day; ^d^Walking included 30–40 min/d at a pace of ∼3 mi/h during all three phases of the study in both groups; ^e^Standard meal included 1 starch, 1, 6 oz. nonfat yogurt (nonfat dairy intake), and 1 fruit; ^f^Variety of flavors provided; ^g^Nonfat yogurt not allowed in the COM group across all phases; ^h^All foods were participant's choice in COM group, foods were participant's choice in INT group, except where noted; ^i^1 cup plain Cheerios, 1 cup Multi-grain Cheerios, 1 Cup Rice-Chex, or 1 Nature Valley Crunchy granola bar (choice of flavors) (General Mills, Inc., Minneapolis, MN); ^j^2 of these were nonfat yogurt.

Intake of calcium and vitamin D were normalized across all ERD patterns in both groups, using the 1700 kcal per day INT diet as the standard, as dietary calcium has been suggested to influence BW (Zemel et al. 2000). Supplementation was designed to match targets of 1500 mg of calcium and 10–20 *μ*g of vitamin D per day (Table1). Investigators dispensed nutrient supplements (Wegmans Calcium Citrate + Vitamin D, Wegmans Food Market, Rochester, NY) to women at weekly educational sessions.

Women in both groups walked 30–40 min per day at a moderate pace (∼3 min/h) during all three phases of the study. Weekly educational sessions were held for both INT and COM groups throughout the 6-month study and included lessons on basic nutrition knowledge, exchange patterns of eating, portion size and control, purchasing and preparing food and modifying recipes as well as motivational lessons on outcome expectations, self-regulation and monitoring, problem-solving, lifestyle modification, emotion eating and motivation for walking. Throughout the 24-week intervention, women in both groups received items, such as insulated lunch bags, measuring cups and food storage containers, to encourage motivation. These items were provided approximately once per month to women. Women were compensated for their time with $20 US after each testing session; women who completed the full study were compensated with an additional $50 US for their time.

### Testing sessions

During this 24-week parallel-arm study, women completed testing sessions (between 0700 and 0930 h) at baseline (pre-intervention) and after 2, 12, and 24 weeks of the intervention. Women fasted for at least 12 h and avoided strenuous physical activity and alcohol intake before each testing session. Women completed 4-day food and physical activity records before each testing session and submitted these records upon arrival. Women completed anthropometric, selected vital signs and body composition measurements. Fasting venous whole blood samples were collected from women at weeks 0, 2, 12, and 24. Whole blood was centrifuged at 1252 × g for 10 min, and serum was aliquotted into cryovials and stored at −80°C for later analysis.

### Dietary and physical activity assessment

Before the study began, participants were trained to accurately complete a 4-day food and physical activity record. To facilitate accuracy in recording of foods and beverages, participants were provided with handouts that contained pictures of common items and standard portion sizes. Training minimized over- and under-reporting by women, as implausible intakes (<500 or >5000 kcal/d) were not reported. Participants were provided with verbal and written directions for the recording of physical activities, using of pedometers and tracking of step counts. Women wore pedometers on their waistbands of clothing, above the hip and aligned with the length of the leg. Pedometers were worn during waking hours, except during showering or bathing, and step counts were recorded for each 24-h period of the 4-day interval. Food and beverage intakes, physical activity, and step counts were recorded during 3 weekdays and 1 weekend day, before each testing session.

Diet records were reviewed with participants upon receipt and verified by a registered dietitian. Records were analyzed for estimated average daily energy (kcal/d), macronutrient (g/d and % kcal/d) and calcium (mg/d) intakes (dietary and supplemental), as well as servings per day of total and nonfat dairy intake, using Nutrition Data System for Research (NDS-R version 2010, Minneapolis, MN) by a registered dietitian blinded to intervention assignment.

Physical activity records were reviewed with participants and used to estimate time (min) spent engaged in light, moderate, and vigorous physical activities, which corresponded to metabolic equivalent levels. Duration of activity, metabolic equivalents, and participant BW were used to calculate total energy expended per day. Data from 4-day records were averaged to produce one estimated average daily energy expenditure value (kcal/d). The number of steps taken by women during each of the 4 days was estimated by pedometers (Accusplit AE120XL, Accusplit Inc., Pleasanton, CA) and averaged to produce one daily estimate. Records were analyzed across all study phases by one research assistant blinded to intervention assignment.

### Anthropometric and body composition measurements

Baseline height was recorded to the nearest 0.1 cm, using a stadiometer (Seca 700, Seca North America East, Hanover, MD). Body weight was measured to the nearest 0.1 kg on a calibrated digital scale (TBF-410GS, Tanita Corporation, Arlington Heights, IL). Participants wore lightweight clothing with bare feet during height and weight measurements. Body mass index was calculated from BW and height. Total body fat mass (kg), fat-free soft tissue mass (kg), body fat percentage, and central abdominal tissue percentage were measured by dual-energy X-ray absorptiometry (Hologic QDR4500A, Bedford, MA). Total body scans were conducted at baseline, week 12 and week 24, by one investigator blinded to intervention assignment. Test–retest reliability in 26 overweight/obese premenopausal women has produced coefficients of variation (CVs) of 1.87, 1.02, and 3.32% for fat mass, fat-free soft tissue mass, and central abdominal tissue percentage, respectively. Waist and hip circumferences were measured in duplicate (Gulik II tape measure, Country Technology, Gays Mills, WI), averaged and recorded to the nearest 0.1 cm.

### Selected vital signs and resting energy expenditure measurements

Resting heart rate (beats/min) of the radial artery was measured by palpation, after a 5-min rest period. Seated systolic and diastolic blood pressure (mm Hg) was measured using a standard sphygmomanometer (Baumanometer® Desk Model, Copiague, NY) after a 5-min rest period. Two measurements were taken for both resting heart rate and blood pressure with a 2- to 3-min rest period between readings, and values were averaged. Resting heart rate and blood pressure measurements were completed by a registered nurse blinded to intervention assignment. Resting energy expenditure was estimated using the Mifflin-St. Jeor equation (Mifflin et al. 1990).

### Biochemical markers of health

Serum was analyzed for triglycerides, total cholesterol, high-density lipoprotein cholesterol, low-density lipoprotein cholesterol, glucose, insulin, leptin, resistin, and adiponectin concentrations. Triglycerides (TG) (mg/dL), total cholesterol (TC) (mg/dL), and high-density lipoprotein cholesterol (HDL-C) (mg/dL) concentrations were measured by spectrophotometry, using standard assay kits (Stanbio Labs, Boerne, TX). Low-density lipoprotein cholesterol (LDL-C) concentration (mg/dL) was calculated using the Friedewald equation: LDL-C = TC − HDL-C − (TG/5) (Friedewald et al. 1972). Serum glucose (mg/dL) was measured by spectrophotometry (Stanbio Labs). Serum insulin (*μ*U/mL) was measured by ultra-sensitive enzyme-linked immunosorbent assay (ALPCO Diagnostics, Salem, NH). Serum leptin (ng/mL), resistin (ng/mL), and adiponectin (*μ*g/mL) were measured by enzyme-linked immunosorbent assay (Bio-Rad, Hercules, CA). Duplicate serum samples were analyzed for each biomarker at corresponding study intervals. Intra- and inter-assay CVs were 2.9 and 6.2% for triglycerides, 2.0 and 4.2% for total cholesterol, and 4.1 and 14.2% for high-density lipoprotein cholesterol, respectively. Intra- and inter-assay CVs were 2.5 and 11.2% for glucose and 4.3 and 12.0% for insulin, respectively. Intra- and inter-assay CVs were 9.3 and 18.7% for leptin, 3.2 and 27.0% for resistin, and 8.4 and 20.9% for adiponectin.

### Statistical analyses

Participants completing the JumpStart phase (*n* = 104) were included in the intention-to-treat analysis. This analysis was conducted using the last observation carried forward approach. All baseline data were normally distributed. Related variables were analyzed by multivariate analysis of covariance with repeated measures on the time factor. Cohort and baseline values were used as covariates in all statistical models. Sample *t*-tests using Bonferroni adjustments for multiple comparisons were completed when significant group or group × time interactions were found. A probability of *P* < 0.01 (two-tailed) was considered statistically significant. Data are reported as means ± standard deviation (SD), unless otherwise noted. Data analyses were conducted using the Statistical Package for the Social Sciences (version 19.0, 2010, SPSS Inc, Chicago, IL). Power analysis using effect size of 0.80 within each group for BW and fat mass in the current study indicated that a sample size of 30 participants per group yielded >0.85 observed power.

## Results

A total of 852 women responded to recruitment methods between July 2009 and September 2010. Of these, 465 women met prescreening criteria (appropriate age, body mass index, and limited self-reported physical activity) and received screening materials, including a Medical History Form, Zung Self-Rating Depression Scale/Status Inventory (Zung 1972) and Informed Consent Form. A total of 321 women returned screening materials which were reviewed by investigators to further determine enrollment eligibility. Of these, 139 women were eligible and obtained medical clearance for participation from their health-care providers.

Baseline testing was completed by 123 healthy premenopausal women with overweight/obesity (Caucasian, *n* = 98; African American, *n* = 11; Other, *n* = 3; No response, *n* = 11). After the JumpStart phase, 104 (INT, *n* = 52; COM, *n* = 52) women (age 33.7 ± 7.3 years; body mass index 29.2 ± 3.1) remained and were included in data analyses (Fig.1). There were no significant differences in baseline age, BW, or body mass index between the 123 women who began the intervention and the 104 women who completed the JumpStart phase (or the 80 women who completed the entire intervention). There were no significant differences in baseline age, BW, or body mass index for participants who completed the intervention (*n* = 80) and participants who withdrew (*n* = 43).

Class attendance was 83% for the INT group and 78% for the COM group. Nonfat yogurt consumption was 73% of prescribed intake in the INT group. Approximately 60% of all women completed the walking protocol on at least 4 days of each week for at least 19 of 24 weeks.

### Dietary and physical activity assessments

Dietary intake data are presented in Table2. Complete food records were available for 71 women. Compared to baseline, estimated average energy intake was reduced by 35.0 and 31.2% for women in the INT and COM groups, respectively, at week 2 and by 25.2 and 30.4% at week 12 and by 18.4 and 27.0% at week 24. As expected, due to study design, estimated energy intake significantly differed between groups only at week 2.

**Table 2:** Estimated energy and macronutrient intakes and energy expenditure from physical activity of premenopausal women with overweight/obesity at baseline and Weeks 2, 12, and 24 in a study examining changes in body composition and metabolic parameters with an energy-reduced dietary pattern (ERD) including moderate protein and increased nonfat dairy intake (INT group) or an ERD with standard protein and standard nonfat dairy intake (COM group), combined with walking

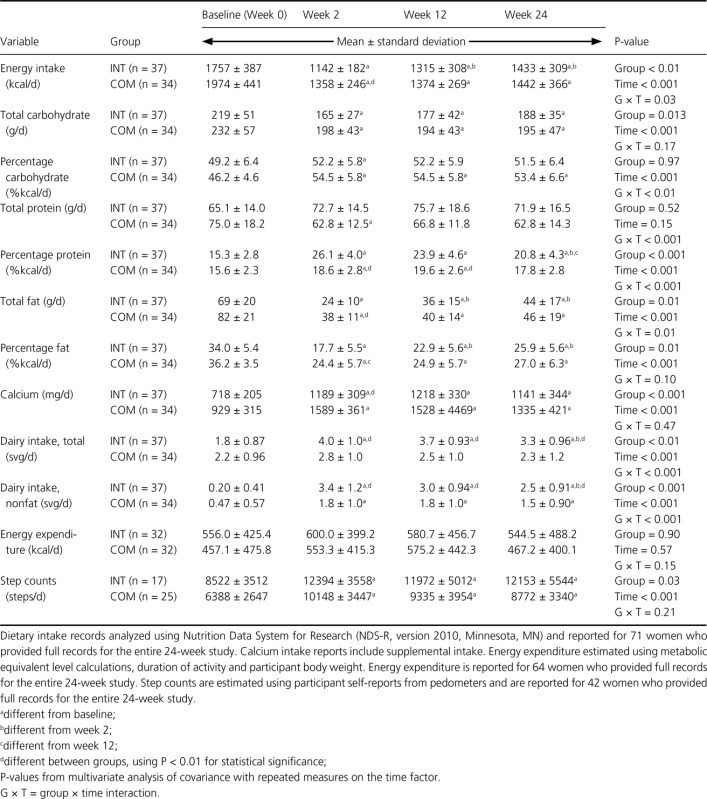

In the INT group, estimated total carbohydrate intake significantly decreased from week 0 to week 2, 12, and 24, while estimated carbohydrate intake as a percentage of total energy intake remained relatively constant, between 49 and 52%. In the COM group, the estimated carbohydrate intake as a percentage of total energy intake significantly increased from baseline to weeks 2, 12, and 24. The estimated protein intake as a percentage of total energy intake increased over time in both groups. Though protein intake did not reach 30% of total energy intake as designed in the INT group, estimated average percentage protein intake was significantly greater in the INT group compared to COM group at week 2 (26.1 vs. 18.6%, respectively, *P* < 0.001) and 12 (23.9 vs. 19.6%, respectively, *P* < 0.001). Both groups reduced the estimated total fat intake and percentage of energy intake as dietary fat over time; the reduction in dietary fat as a percentage of total energy intake was significantly different between groups only at week 2.

Servings of total (*P* < 0.01) and nonfat (*P* < 0.001) dairy intake were greater in the INT group at weeks 2, 12, and 24 compared to the COM group. The estimated total and nonfat dairy intake significantly increased from baseline to weeks 2, 12, and 24 in the INT group. The estimated nonfat dairy intake significantly increased from baseline to weeks 2, 12, and 24 in the COM group. Mean calcium intakes significantly increased from baseline to weeks 2, 12, and 24 in both groups. The calcium intake was greater (*P* < 0.001) in the COM group at week 2 compared to the INT group.

Energy expenditure from physical activity data are presented in Table2. Complete physical activity records were available for 64 women, and complete pedometer step counts were available for 42 women. There was no effect of the diet group or time on the estimated energy expenditure from physical activity. There was no effect of the diet group on the estimated number of step counts. Both groups significantly increased estimated step counts from baseline at weeks 2, 12, and 24.

### Anthropometric and body composition measurements

There was no significant effect of diet group on body composition or anthropometric measurements (Table3). Women in both groups significantly reduced BW, body mass index, and waist and hip circumferences during the JumpStart phase. Women in the INT and COM groups had significant decreases in BW (−4.9 ± 3.2 and −4.3 ± 3.3 kg), body mass index (−1.8 ± 1.2 and −1.6 ± 1.2 kg/m^2^), fat mass (−3.0 ± 2.2 and −2.3 ± 2.3 kg), fat-free soft tissue mass (−1.5 ± 1.6 and −1.7 ± 2.1 kg), body fat percentage (−1.7 ± 1.7 and −1.2 ± 2.0%), waist circumference (−4.4 ± 2.9 and −3.8 ± 3.1 cm), hip circumference (−4.7 ± 3.5 and −4.3 ± 3.6 cm), and central abdominal tissue percentage (−3.0 ± 3.2 and −2.8 ± 2.9%), respectively, from baseline to week 12. These losses were maintained from week 12 through week 24. A group × time interaction was not observed for any of these outcome variables.

**Table 3:** Anthropometric and body composition measurements of premenopausal women with overweight/obesity at baseline and weeks 2, 12, and 24 in a study examining changes in body composition and metabolic parameters with an energy-reduced dietary pattern (ERD) including moderate protein and increased nonfat dairy intake (INT group) or an ERD with standard protein and standard nonfat dairy intake (COM group), combined with walking

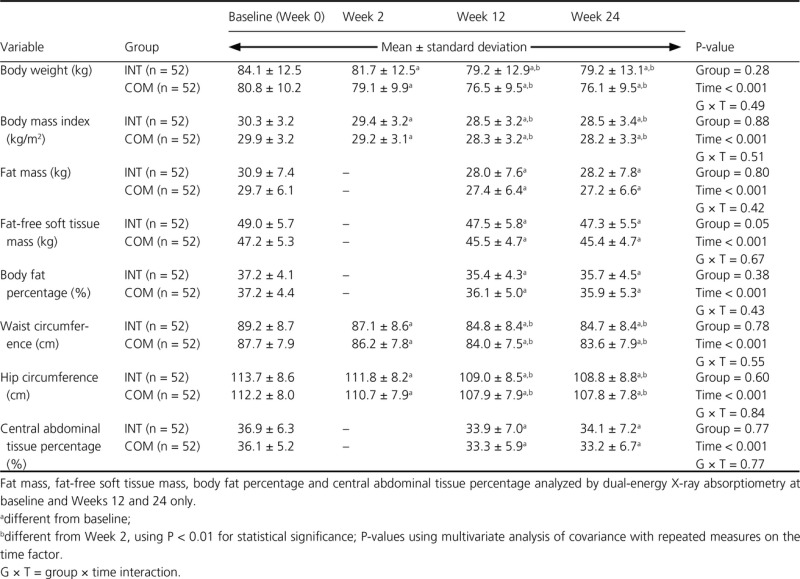

### Selected vital signs and resting energy expenditure measurements

There was no significant effect of the diet group on resting heart rate, systolic, or diastolic blood pressure, or resting energy expenditure (Table4). There were no significant differences in resting heart rate or systolic or diastolic blood pressure over time in either group. A group × time interaction was not observed for any of these measures. There was a significant reduction in resting energy expenditure from baseline to week 12, and this reduction was maintained through week 24.

**Table 4:** Resting heart rate, blood pressure and resting energy expenditure measurements of premenopausal women with overweight/obesity at baseline and weeks 2, 12, and 24 in a study examining changes in body composition and metabolic parameters with an energy-reduced dietary pattern (ERD) including moderate protein and increased nonfat dairy intake (INT group) or an ERD with standard protein and standard nonfat dairy intake (COM group), combined with walking

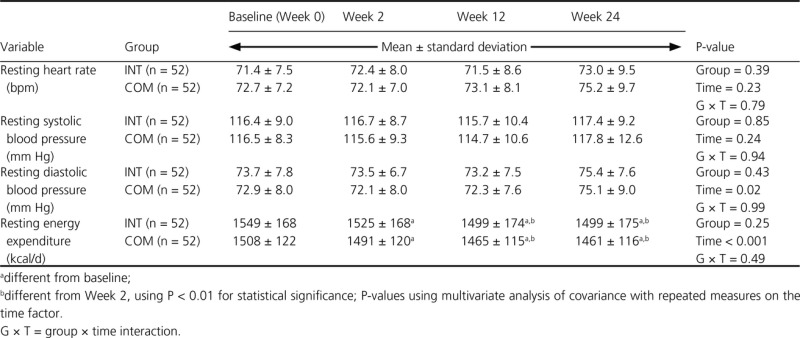

### Biochemical markers of health

There were no significant diet group effects for biochemical markers of health (Table5). Women in the INT and COM groups experienced significant reductions in triglycerides and total cholesterol concentrations at week 12 that were maintained to week 24. Serum low-density lipoprotein cholesterol significantly decreased at week 12, but this reduction was no longer significant at week 24. There were no other significant changes in groups over time, and a group × time interaction was not observed in any of these secondary outcome measures.

**Table 5:** Blood lipid, glucose and insulin concentrations of premenopausal women with overweight/obesity at baseline and weeks 2, 12, and 24 in a study examining changes in body composition and metabolic parameters with an energy-reduced dietary pattern (ERD) including moderate protein and increased nonfat dairy intake (INT group) or an ERD with standard protein and standard nonfat dairy intake (COM group), combined with walking

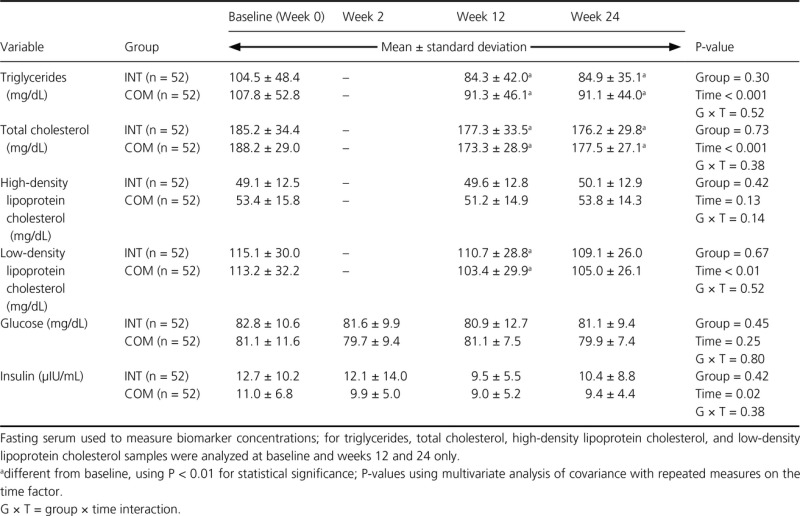

A significant diet group effect was not found for serum leptin, resistin, or adiponectin concentrations (Table6). Leptin concentration significantly decreased in both groups at week 12, and this reduction was maintained through week 24. There were no significant changes in resistin concentration over time for either group. There was a significant increase in adiponectin between weeks 12 and 24 in both groups. Significant group × time interactions were not observed in these adipose-derived hormones.

**Table 6:** Concentrations of adipose-derived hormones in premenopausal women with overweight/obesity at baseline and Weeks 12 and 24 in a study examining changes in body composition and metabolic parameters with an energy-reduced dietary pattern (ERD) including moderate protein and increased nonfat dairy intake (INT group) or an ERD with standard protein and standard nonfat dairy intake (COM group), combined with walking

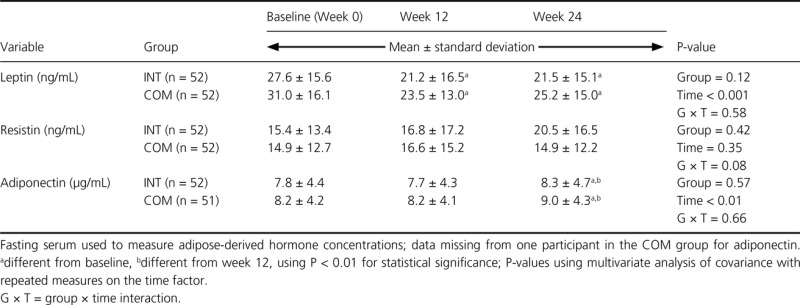

## Discussion

Healthy premenopausal women with excess adiposity significantly reduced BW and fat mass as well as triglycerides and total cholesterol across a 12-week JumpStart + weight-loss phase and maintained these changes across a 12-week weight-loss maintenance phase of a lifestyle intervention, including diet modification and walking. The lack of detectable differences in main outcome variables between diet groups at week 12 suggests that these two approaches to BW loss may have been equally effective. Both interventions significantly increased the number of nonfat dairy servings participants were consuming per day and met recommendations for protein intake within the acceptable macronutrient distribution range (National Academies Press 2005). Although protein comprised a greater proportion of total energy in the INT group, estimated dietary protein intake did not reach the goal of 30% of total energy and fell within the range of 0.89–0.96 g of protein per kg BW per day. Estimated protein intake in the COM group was within the range of 0.78–0.87 g of protein per kg BW per day. Absolute protein (g) consumed during energy restriction is often less than the amount consumed during energy balance, though protein may comprise the same percentage of overall energy intake (Pasiakos et al. 2014). During energy restriction, grams of protein intake per kg BW may fall below 0.8. Within the context of an ERD (i.e., lowered total energy intake), dietary protein as a percentage of total energy should increase to meet a minimally recommended amount (National Academies Press 2005).

In order to maintain fat-free soft tissue mass, protein intake should increase or at least be maintained as BW declines due to reduced overall caloric intake (Layman 2004). Interventions citing improved body composition and blood lipid outcomes within experimental higher-protein diet groups compared to standard-protein diet groups with or without exercise often include dietary protein at 1.3–1.6 g of protein per kg BW per day (approximately 30% of the total energy consumed; Layman et al. 2003b, 2005; Leidy et al. 2007; Meckling and Sherfey 2007; Josse et al. 2011). This level of protein intake was not met in the current study, likely due to the study design leveraging a dietary exchange weight-loss program in free-living women. Studies that provide meals or utilize controlled feedings may better achieve a strict dietary protein goal.

Participants in both groups similarly improved triglycerides and total cholesterol irrespective of dietary intervention, indicating overall improvement in these metabolic parameters with energy restriction, moderate walking and BW loss (Blumenthal et al. 2010). This is in contrast to other studies in which larger reductions in triglycerides concentrations were found in participants with greater protein intake (Skov et al. 1999; Farnsworth et al. 2003; Layman et al. 2003b, 2005, 2009; Noakes et al. 2005). However, these previous studies differed in research design and participant characteristics and compared wider extremes of dietary protein intake than in the current study. Protein as a percentage of total energy intake differed by approximately 12% between intervention groups in previous studies (Skov et al. 1999; Farnsworth et al. 2003; Layman et al. 2003b, 2005, 2009; Noakes et al. 2005), while the current study achieved an approximate 5% difference in dietary protein intake between groups. The difference in carbohydrate intake between the INT and COM groups was roughly two starch servings at week 2 and one at week 12, with no appreciable difference at week 24. This suggests that an approximate 2% difference in dietary carbohydrate intake between groups was insufficient to impact secondary outcomes. Systolic and diastolic blood pressure remained unchanged and within normal blood pressure ranges (Goldstein et al. 2011).

In the current study, the JumpStart phase was designed to accelerate BW loss within the INT group. Interventions show that a more substantial initial BW loss (within the first 5–12 weeks) predicts overall BW loss (Wadden et al. 1992; Guaraldi et al. 2011) and maintenance of BW loss over time (Wadden et al. 1992; Jeffery et al. 1998; Stotland and Larocque 2005; Guaraldi et al. 2011) provided that early BW loss is significant and followed by lifestyle changes that are monitored over time (Toubro and Astrup 1997; Astrup and Rössner 2000). Estimated energy intake was significantly lower in the INT group compared to the CON group, and though not significant at *P* < 0.01 in the context of the entire intervention, BW (and body mass index) was reduced to a greater extent (*P* < 0.01) in the INT group than the COM group at Week 2 (−2.4 ± 1.0 vs. −1.7 ± 1.1 kg). The 2-week JumpStart phase may not have been sufficient in length to induce significant differences in BW loss (at *P* < 0.01) between groups as part of the entire 12-week weight-loss phase plus 12-week weight-loss maintenance phase (Wadden et al. 1992; Stotland and Larocque 2005; Guaraldi et al. 2011).

Mechanisms for improved metabolic conditions with BW loss may be partially explained by beneficial changes in select adipose-derived hormones (Jung et al. 2008). Leptin, resistin, and adiponectin originate predominately in adipose tissue (Rajala and Scherer 2003; Klempel and Varady 2011) and are known to contribute to the inflammatory milieu (Ma et al. 2012). While leptin and resistin are positively correlated with body mass index (Friedman 2002; Pagano et al. 2005), low adiponectin levels are associated with obesity (Weyer et al. 2001). In their dysregulated states, these adipose-derived hormones may negatively impact other body systems. Indeed, adipose tissue-derived hormones have been implicated in the progression of cardiometabolic disease (Allende-Vigo 2010). In the current study, reductions in leptin and increases in adiponectin are consistent with other studies demonstrating BW and fat mass loss (Miller et al. 2009; Varady et al. 2009; Josse et al. 2012). A review of adipose-derived hormone response to randomized control energy-restricted diets reports that fat mass losses of <10% may be insufficient to reduce circulating resistin and increase adiponectin levels; however, studies examining resistin response to BW and fat mass loss are limited (Klempel and Varady 2011). Leptin is particularly responsive to BW loss induced by energy restriction (Klempel and Varady 2011), responding to both short term alterations in energy intake (Sartorio et al. 2003) and reductions in fat mass. Further insight into the function of these adipose-derived hormones and their part in the improvement of metabolic factors observed during BW and fat mass loss should be a research priority.

Strengths of the current study include using a research design with free-living women to examine the effectiveness of the intervention (Most et al. 2003). Findings from this study may be directly generalisable to women of similar characteristics and relevant regarding the challenges faced by women who attempt this approach to BW loss. Additionally, this study included a daily moderate walking component in both groups. As an accepted promoter of BW and fat mass losses with energy restriction (Jakicic and Davis 2011), increased physical activity was not among study variables but a component of the overall intervention strategy. Similarly, all women attended weekly nutrition-related educational sessions that did not differ between groups, as attendance at group sessions during weight-loss intervention has been strongly associated with weight loss (Sacks et al. 2009). This study employed a comparison group following a dietary pattern adequate in both protein and dairy/calcium, rather than inadequate in dairy/calcium as evaluated in past studies (Zemel et al. 2004, 2005). In addition, the current study employed a comparison group meeting protein and dairy/calcium recommendations for evaluation against the experimental diet, thereby examining effects of protein and dairy/calcium intake beyond general recommendations. Studies reporting that dairy intake or calcium supplementation may accelerate BW and fat mass loss in the context of energy restriction often begin with participants who are calcium deficient or use a low-calcium, energy-restricted control group (Zemel et al. 2004, 2005; Josse et al. 2011). Recruitment in the current study was not limited to women who were deficient in either protein or dairy intakes, making this design more generalisable. Additionally, calcium intake was controlled via supplementation. Finally, this study used highly conservative statistical methods, limiting the likelihood of type 1 error, though this may have inflated the rate of false negatives.

This study had several limitations. Studies involving free-living participants allow for more realistic examinations of intervention components (Most et al. 2003); however, the lack of complete food intake control in the current study limited the ability to address diet efficacy on BW and body composition measurements. For example, protein intake goals for the INT group were not achieved, likely due to the lack of a feeding trial study design. Additionally, self-reported physical activity and pedometer counts are prone to error (Melanson et al. 2004; Ferrari et al. 2007). Clearly, many women elected not to complete the walking component of the intervention. Generalizations from this study may be limited to only healthy, premenopausal and primarily Caucasian women.

In conclusion, an ERD including moderate protein and increased nonfat dairy combined with walking exercise resulted in significant losses in BW and fat mass along with other beneficial changes in anthropometric measurements and some metabolic biomarkers in healthy premenopausal women with excess adiposity. An ERD that meets dietary protein (0.8 g protein/kg BW) and dairy intake (3 servings/d) recommendations is as effective as an ERD that includes greater protein and more servings of nonfat dairy intake per day in producing BW and fat mass losses and maintaining these beneficial changes over time. Additionally, the current study did not demonstrate any benefit of early BW loss on longer-term BW loss and maintenance of that BW loss. Either dietary approach to BW loss and maintenance used in this study may be applied in clinical practice with modest outcomes.
